# Effects of magnesium citrate, magnesium oxide and magnesium sulfate supplementation on arterial stiffness in healthy overweight individuals: a study protocol for a randomized controlled trial

**DOI:** 10.1186/s13063-019-3414-4

**Published:** 2019-05-28

**Authors:** Joëlle C. Schutten, Peter J. Joris, Ronald P. Mensink, Richard M. Danel, Frans Goorman, M. Rebecca Heiner-Fokkema, Rinse K. Weersma, Charlotte A. Keyzer, Martin H. de Borst, Stephan J. L. Bakker

**Affiliations:** 1 Department of Internal Medicine, Division of Nephrology, University of Groningen, University Medical Center Groningen, Hanzeplein 1, 9700RB, Groningen, the Netherlands; 2 Department of Nutrition and Movement Sciences, NUTRIM School of Nutrition and Translational Research in Metabolism, Maastricht University Medical Center, Maastricht, the Netherlands; 3 Magnesium Health Institute, Groningen, the Netherlands; 4 Nedmag B.V, Veendam, the Netherlands; 5 Department of Laboratory Medicine, Laboratory of Metabolic Diseases, University of Groningen, University Medical Center Groningen, Groningen, the Netherlands; 6 Department of Gastroenterology and Hepatology, University of Groningen, University Medical Center Groningen, Groningen, the Netherlands

**Keywords:** Magnesium supplements, Arterial stiffness, Blood pressure, Gut microbiota, Randomized controlled trial

## Abstract

**Background:**

Arterial stiffness is closely related to the process of atherosclerosis, an independent cardiovascular risk factor, and predictive of future cardiovascular events and mortality. Recently, we showed that magnesium citrate supplementation results in a clinically relevant improvement of arterial stiffness. It remained unclear whether the observed effect was due to magnesium or citrate, and whether other magnesium compounds may have similar effects. Therefore, we aim to study the long-term effects of magnesium citrate, magnesium oxide and magnesium sulfate on arterial stiffness. In addition, we aim to investigate possible underlying mechanisms, including changes in blood pressure and changes in gut microbiota diversity.

**Methods:**

In this randomized, double-blind, placebo-controlled trial, a total of 162 healthy overweight and slightly obese men and women will be recruited. During a 24-week intervention, individuals will be randomized to receive: magnesium citrate; magnesium oxide; magnesium sulfate (total daily dose of magnesium for each active treatment 450 mg); or placebo. The primary outcome of the study is arterial stiffness measured by the carotid–femoral pulse wave velocity (PWV_c–f_), which is the gold standard for quantifying arterial stiffness. Secondary outcomes are office blood pressure, measured by a continuous blood pressure monitoring device, and gut microbiota, measured in fecal samples. Measurements will be performed at baseline and at weeks 2, 12 and 24.

**Discussion:**

The present study is expected to provide evidence for the effects of different available magnesium formulations (organic and inorganic) on well-established cardiovascular risk markers, including arterial stiffness and blood pressure, as well as on the human gut microbiota. As such, the study may contribute to the primary prevention of cardiovascular disease in slightly obese, but otherwise healthy, individuals.

**Trial registration:**

ClinicalTrials.gov, NCT03632590. Retrospectively registered on 15 August 2018.

**Electronic supplementary material:**

The online version of this article (10.1186/s13063-019-3414-4) contains supplementary material, which is available to authorized users.

## Background

Magnesium is an essential mineral that acts as a cofactor in hundreds of enzymatic reactions in the human body. It is, therefore, not surprising that insufficient magnesium intake has been associated with a wide variety of metabolic disorders, such as insulin resistance, and common diseases that may result from metabolic deregulations, such as hypertension and ischemic heart disease [1–5]. Foods with relatively high magnesium contents include whole grains, leafy green vegetables, legumes and nuts.

Arterial stiffness is closely related to the extent of atherosclerosis [6], an independent cardiovascular risk factor [7], and predictive of future cardiovascular events and mortality [8]. The gold standard for quantifying arterial stiffness is the carotid–femoral pulse wave velocity (PWV_c–f_), a noninvasive method that measures the propagation of the forward pressure wave traveling along the aorta [8]. Recently, we observed a significant reduction of arterial stiffness, measured by the PWV_c–f_, after 24 weeks of oral magnesium citrate supplementation (total daily dose of 350 mg) in the magnesium citrate group compared with the placebo group (8.3 m/s vs 9.1 m/s, respectively) [9]. Although blood pressure is a major determinant of arterial stiffness [10], we did not observe any change in blood pressure. This lack of effect on blood pressure could be due to the total daily dose, which may have been too low to detect changes in blood pressure. A meta-analysis by Kass *et al.* [11] indeed showed that supplementation of > 370 mg/day resulted in a more pronounced blood pressure-lowering effect. In addition, we could not differentiate whether the beneficial effect on arterial stiffness was due to the supplementation of magnesium or due to citrate. The mechanism by which magnesium decreases arterial stiffness is not entirely known, but an effect on gut microbiota has been observed [12–14]. Previous studies have shown that magnesium deficient diets resulted in an altered microbiome and, more specifically, decreased microbial diversity in mice [13, 15, 16]. Gut microbiome diversity has recently been shown to be inversely associated with arterial stiffness, independent of obesity-related traits, such as insulin and visceral fat [17].

The primary aim of the current study is to replicate the effect of magnesium citrate on arterial stiffness that we found in our previous study. Secondly, we aim to test whether magnesium oxide and magnesium sulfate are noninferior to the effect of magnesium citrate on arterial stiffness and to unravel possible underlying mechanisms, including changes in blood pressure and gut microbiota diversity and metabolites.

## Methods/design

### Trial design

**Fig. 1:**
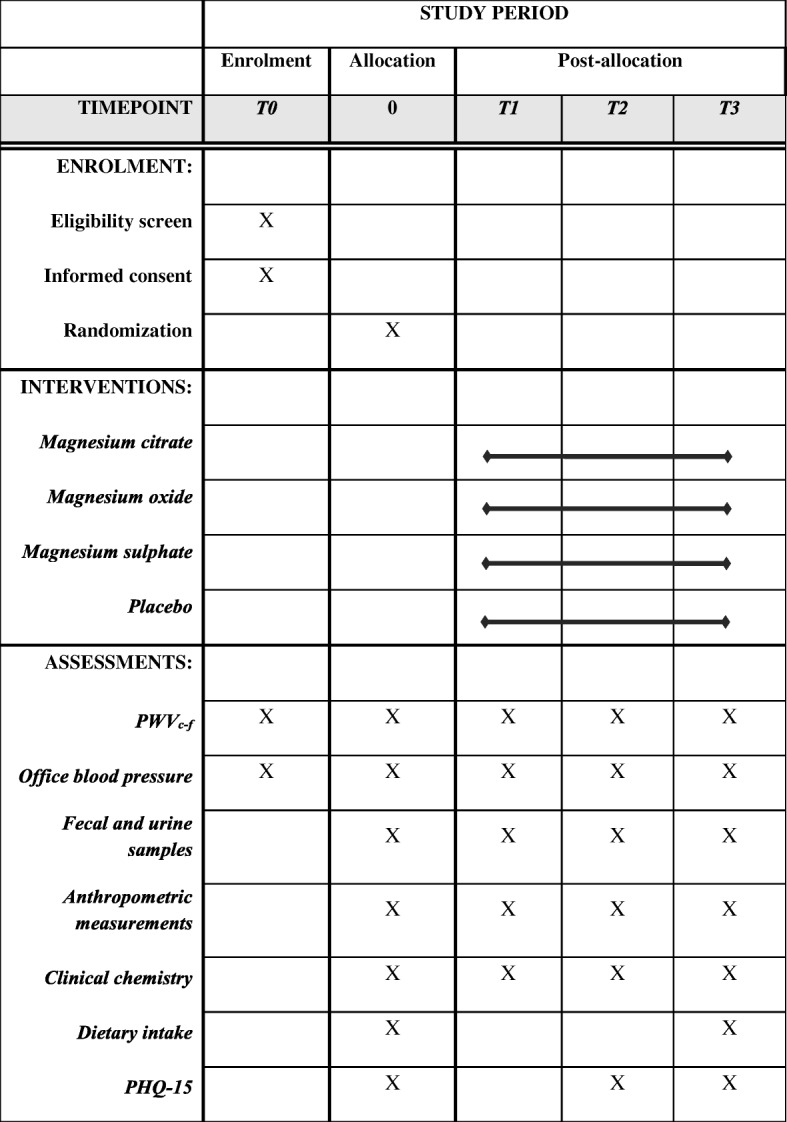
Schedule of enrolment, interventions and assessment. PWV_c–f_ carotid-to-femoral pulse wave velocity, PHQ-15 Patient Health Questionnaire

In this randomized, double-blind, placebo-controlled trial, healthy overweight and slightly obese volunteers will be allocated to one of the three magnesium groups or the placebo control group. This study will be conducted at the University Medical Center Groningen (UMCG). The current protocol was written in accordance with the Standard Protocol Items: Recommendations for Interventional Trials (SPIRIT) guideline (Fig. 1 and Additional file 1) [18]. Ethics committee approval was obtained from the UMCG, the Netherlands.

### Characteristics of participants

For the present study, only subjects who provide written informed consent are eligible. Furthermore, participants should be between 45 and 75 years and should be overweight or slightly obese (body mass index between 25 and 35 kg/m^2^), because these subjects are expected to have increased arterial stiffness and blood pressure at baseline, allowing for improvement by the intervention [19]. To avoid any possible variations in the study outcomes due to hormonal effects, only postmenopausal women (≥ 2 years after last menstruation) will be included. Participants who meet any of the following criteria are not eligible for this study:

- high magnesium intake (defined as urinary magnesium excretion of ≥ 7.0 or ≥ 5.9 mmol/24 h for men and women, respectively);
- plasma glucose ≥ 7.0 mmol/l;
- serum total cholesterol ≥ 8.0 mmol/l;
- serum triglycerides ≥ 2.2 mmol/l;
- current smoker or smoking cessation < 12 months;
- presence of diabetes mellitus;
- familial hypercholesterolemia;
- abuse of drugs;
- more than 21 alcoholic consumptions per week;
- unstable body weight (weight gain or loss > 3 kg in the past 3 months);
- use of proton pump inhibitors;
- use of lipid-lowering drugs and/or antihypertensive therapy that started in the past 6 months;
- use of magnesium supplements or an investigational product within another biomedical within the previous 1 month;
- severe medical conditions that might interfere with the study, such as epilepsy, asthma, kidney failure or renal insufficiency, chronic obstructive pulmonary disease, inflammatory bowel diseases, autoinflammatory diseases and rheumatoid arthritis;
- active cardiovascular disease; or
- not willing to give up being a blood donor (or having donated blood) from 8 weeks before the start of the study and during the study.

### Interventions

**Fig. 2:**
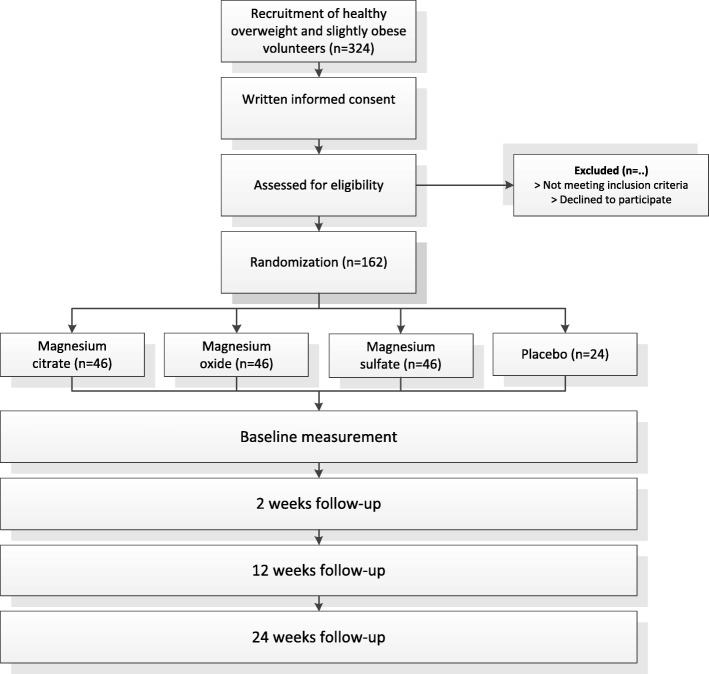
Flow chart of the study design

The overweight and slightly obese participants will be randomly assigned to receive: magnesium citrate; magnesium oxide; magnesium sulfate; or placebo. A flow chart of the study interventions is presented in Fig. 2. Study participants will be requested to take 2 capsules thrice daily for 24 weeks. On the test days, the first 2 capsules will be taken after all measurements are completed. Each magnesium supplement provides 75 mg magnesium (Magnesium Citrate Complex (Mg 15.5%) Magnesium Sulphate Complex (17.4%) and Magnesium Oxide Complex (60.3%); AMT Laboratories Inc.). Thus, daily magnesium intake provided by the 6 capsules will be 450 mg. The placebo capsules will contain starch (Amylum solani). The total treatment duration of 24 weeks is based on our previous study that showed a significant effect of magnesium supplementation on arterial stiffness after 24 weeks [9]. At baseline and weeks 2, 12 and 24, participants will undergo PWV_c–f_, office blood pressure and body composition measurements. Prior to each test day, participants will collect 24-h urine and fecal samples. In addition, participants will be requested to complete the Patient Health Questionnaire (PHQ-15) before each test day. The PHQ-15 is a somatic symptom subscale derived from the full PHQ. This questionnaire includes about 15 somatic symptoms or symptom clusters that account for more than 90% of the physical complaints reporting in the outpatient setting [20]. Finally, subjects will record any signs of illness in diaries, as well as medication used, and any deviations from the protocol during the study. Subjects are requested not to change their physical activity level, their food intake pattern or use of alcohol during the entire study.

### Blinding and randomization

To ensure the double-blind design, optically similar capsules will be packed and coded by laboratory Medisan (Heerenveen, the Netherlands), who will provide the study medication (magnesium supplements and placebo). In addition, this laboratory will conduct the randomization procedure. For this, they will create a categorical list in logical order. This list will include one intervention for each subject. Based on a computer-generated list of random number, subjects will be randomized into one of the groups. This results in a randomized list of treatment allocation.

### Sample size calculation

The current study is powered on arterial stiffness, the primary endpoint. The required sample size for noninferiority testing is calculated based on a margin of 0.5 m/s, a probability of 0.05 and a power of 0.80. Therefore, 41 subjects per magnesium group are needed. To compare magnesium citrate with placebo, we assume that the number of subjects per group calculated for a two-arm trial (*n* = 24) is sufficient. Because a 10% dropout rate is expected, 162 will be recruited to start the study. By taking into account that only 50% of the subjects included in the screening will meet all inclusion criteria and are willing to participate, 324 subjects are expected to participate in the screening visits.

This sample size calculation is based on an intra-subject variability of 0.9 m/s and a 1.0 m/s change in the PWV_c–f_ after 24 weeks of magnesium supplementation found in our previous study [9]. The equivalence margin of 0.5 m/s is based on the observed effect [9], which is 50% of the magnesium citrate supplementation effect compared to placebo. For the calculation of the sample size, the formula of Julious [21] was used.

### Compliance to the intervention

Compliance will be assessed based on the number of returned capsules after 12 and 24 weeks. Subjects will be considered compliant when they take at least 80% of the capsules. In addition, we will assess compliance by urinary magnesium concentrations at the end of the study.

### Main outcome

The PWV_c–f_ will be recorded in duplicate using the SphygmoCor v9 (AtCor Medical, West Ryde, Australia). First, the distance from the suprasternal notch to the femoral recording site (via umbilicus) will be measured [22]. Subsequently, the distance from the suprasternal notch to the carotid recording site (right) will be subtracted. In addition, the direct distance between the carotid recording site and the femoral recording site (right) will be measured. Second, ECG electrodes will be applied in order to identify the R-wave in the ECG complex. Then, using the tonometer, the arrival of the pulse wave and the delay to the R-wave of the ECG will be measured at the carotid and femoral arteries. If all correct measures are entered and recorded, the program of the manufacturer will calculate the PWV_c–f_ automatically. Using the same tonometer applied to the radial artery near the wrist of the right arm, pulse wave analysis (PWA) will be determined. Measurements are synchronized beat to beat and will be recorded in duplicate. From these recordings, the program of the manufacturer will determine the central augmentation index adjusted for heart rate (CAIxHR75) automatically.

### Secondary outcomes

#### Blood pressure

Office blood pressure (systolic and diastolic) and heart rate will be monitored using a continuous blood pressure monitoring device (Criticare 506 N3; Criticare Systems Inc., Waukesha, WI, USA). The investigator will measure the blood pressure before the PWV_c–f_ measurements. After an acclimatization period of at least 15 min in the supine position, office blood pressure will be measured. The first measurement will be discarded, and the mean of the last three measurements will be reported [23].

#### Gut microbiota

Subjects will be asked to collect a fecal sample 1 or 2 days prior to the test day and store the sample in the fridge at home. They will be asked to bring the sample to the UMCG on ice (cooling elements or in a bag with ice cubes). On the test day, the samples will be immediately stored at − 80 °C. We will investigate the microbiome diversity, specific operational taxonomy as well as specific circulating metabolites, including phenylacetylglutamine, trimethylamine oxide (TMAO) and indoleproprionate (IPA), which have been previously linked to cardiovascular events and metabolic syndrome, respectively [24, 25]. The composition of the gut microbiota in fecal samples will be analyzed with PCR amplification of the 16S rRNA V4–V5 variable region and deep sequencing using the Illumina® MiSeq platform.

#### Anthropometric measurements

On each test day, anthropometric measurements (body weight without shoes and heavy clothing and body composition) will be performed to characterize participants. Body composition (e.g. fat mass, lean body mass, extracellular water and intracellular water) will be measured by Bio-Electrical Impedance Analysis (Bodystat Quadscan 4000®; Quadscan, Douglas, Isle of Man, UK). The measurements will be carried out with the patient in the supine position for 5 min, with their arms parallel to and separated from the trunk and their legs apart. The electrode sites will be cleaned with an alcohol wipe. Two electrodes will be placed on the right hand and wrist, and another two on the right foot and ankle.

#### Patient Health Questionnaire

Participants will be requested to complete the Patient Health Questionnaire (PHQ-15) before each test day. The PHQ-15 is a somatic symptom subscale derived from the full PHQ. This questionnaire includes about 15 somatic symptoms or symptom clusters that account for more than 90% of the physical complaints reporting in the outpatient setting [20].

#### Clinical chemistry

Blood samples will be analyzed at the Department of Laboratory Medicine of the UMCG, according to routine procedures: plasma levels of magnesium, sodium, potassium, chloride, calcium, phosphate, urea, total protein, albumin, creatinine and hemoglobin, hematocrit, leukocytes, thrombocytes and metabolic risk markers (triglycerides, total cholesterol, LDL-cholesterol and HDL-cholesterol, fasting plasma glucose and HbA1C) will be measured. In addition, urinary calcium, sodium, potassium, chloride and creatinine will be determined. To determine the magnesium status, plasma magnesium levels and intra-erythrocyte magnesium will be measured according to routine procedures.

#### Dietary intake

To ensure that participants have maintained their food pattern during the study, subjects are asked to report their diet for 3 consecutive days at the start and at the end of the study in dietary diaries using standard supply units [26]. These diaries will be checked for completeness by the investigator.

### Statistical analysis

For normally distributed parameters, data will be presented as the mean value and standard deviation. Nonnormally distributed parameters will be presented as the median (range). For statistical analyses, responses will be calculated defined as the difference between follow-up and baseline values. For the primary analyses, in which the effect of magnesium citrate on arterial stiffness will be evaluated, differences in responses between magnesium citrate and placebo will be tested (one-factor ANCOVA) with use of the baseline measurements of the outcome variables as covariates. In order to test as secondary analyses whether the effect of magnesium oxide and magnesium sulfate on arterial stiffness are noninferior to the effect of magnesium citrate on arterial stiffness, a noninferiority margin (Δ) of 0.5 m/s is chosen based on the effect size of magnesium citrate on arterial stiffness found in previous research [9]. A one-factor ANCOVA, with use of the baseline measurements of the outcome variables as covariates, will be conducted to evaluate differences in responses between magnesium and placebo treatments. To prove noninferiority, the lower limit of the confidence interval of the difference between magnesium sulfate and magnesium citrate and the difference between magnesium oxide and magnesium citrate must not exceed the limit of Δ to be 95% sure that magnesium oxide and magnesium sulfate are not worse than magnesium citrate supplements by more than Δ. Treatment effects will be calculated at each time point (after 2, 12 and 24 weeks) with one-factor ANCOVA. Differences are considered statistically significant at *P* < 0.01 for the primary analyses (confirmatory) and *P* < 0.05 for the secondary analyses (exploratory). Statistical analyses will be performed using SPSS 23.0 software for Windows (SPSS Incorporated, Chicago, IL, USA). Normality of the residuals will be examined using the Shapiro–Wilk normality test. If a residual is not normally distributed, parameters will be log-transformed. If after log-transformation the residuals are still not normally distributed, nonparametric tests will be used. Missing data will not be replaced by estimates. Because of randomization, it is expected that confounders will be divided equally. Therefore, no adjustment for confounding will take place. The intention-to-treat principle will be applied in primary analyses. Secondly, we will conduct per-protocol analyses (exclude noncompliance subjects from the statistical analysis). To prove noninferiority, the confidence interval of the difference must exclude the margin in both the intention-to-treat and the per-protocol analyses. Finally, we will conduct subgroup analyses in which we will compare the effects of magnesium on arterial stiffness in subjects on antihypertensive therapy and subjects not on antihypertensive therapy. Because over time arterial stiffness may increase more rapidly in subjects treated for hypertension, our hypothesis is that magnesium may have a more pronounced effect on arterial stiffness in subjects on antihypertensive therapy.

## Discussion

The current study aims to replicate the effect of magnesium citrate on arterial stiffness that we found in our previous study [9], now with a slightly higher dose and to evaluate whether magnesium oxide and magnesium sulfate are noninferior to the effect of magnesium citrate in terms of arterial stiffness. We furthermore aim to unravel possible underlying mechanisms, including changes in blood pressure and gut microbiota diversity. To our knowledge, this is the first clinical trial that compares the long-term effects of different commercially available magnesium supplements on well-established cardiovascular risk markers, including arterial stiffness, blood pressure and gut microbiota [27–29]. Results of longitudinal epidemiologic studies have found that a decrease of 1.0 m/s in arterial stiffness, measured by the PWV_c–f_, corresponded to a reduced risk of cardiovascular events of 14% [30], underlining the potential clinical relevance of magnesium supplementation in the prevention of cardiovascular disease.

So far, only three studies have addressed the effect of magnesium supplementation on arterial stiffness [9, 31, 32]. Cunha *et al*. [31] reported an effect on blood pressure and endothelial function in hypertensive women after magnesium supplementation. However, no effect on arterial stiffness was found. Furthermore, the study by Cosaro *et al.* [32] showed neither an effect on blood pressure nor on arterial stiffness. Of note, the magnesium formulation, total daily dose, trial duration and study design were different between these studies. No studies have performed a head-to-head comparison between various magnesium formulations. Particularly, the difference between organic and inorganic formulations may be of interest.

It has been suggested that organic magnesium formulations (e.g. magnesium citrate) are superior to inorganic magnesium formulations, such as magnesium oxide, in terms of bioavailability [33, 34]. However, a meta-analysis of 34 clinical trials demonstrated that individuals supplemented with inorganic compounds, mainly with chloride as the counterbalancing anion, showed higher serum magnesium levels and more pronounced blood pressure reduction compared to organic compounds [35]. A total daily dose of 450 mg was chosen, because previous clinical trials showed a greater blood pressure-lowering response when the total daily dose of magnesium was increased [35].

In conclusion, the present study is expected to provide evidence for the effects of different commercially available magnesium formulations (organic and inorganic) on well-established cardiovascular risk markers, including arterial stiffness and blood pressure, as well as on the human gut microbiota. The total daily dose of magnesium in the current study is based on previous meta-analyses that reported greater efficacy at higher dosages. Based on dietary recommendations and current evidence, together with the good safety profile of magnesium supplements and the low rate of adverse events so far reported by previous studies, no risks are expected after a daily supplement of 450 mg in the form of magnesium citrate, magnesium oxide and magnesium sulfate.

### Trial status

The trial is ongoing. Approximately 100 subjects have been included in the study at the time of submission. Additional subjects needed for the study will be recruited by advertisements in newspapers. The recruitment of participants for this study began in September 2017, and is presently ongoing. Recruitment is expected to be completed by the end of 2019.

## Additional file

Additional file 1: SPIRIT 2013 Checklist: Recommended items to address in a clinical trial protocol and related documents (DOC 120 kb)

## Data Availability

Not applicable.

## Abbreviations

ANCOVA: Analysis of covariance
PWA: Pulse wave analysis
PWV_c–f_: Carotid–femoral pulse wave velocity
SPIRIT: Standard Protocol Items: Recommendations for Interventional Trials
UMCG: University Medical Center Groningen
